# *Acinetobacter* Strain KUO11TH, a Unique Organism Related to *Acinetobacter pittii* and Isolated from the Skin Mucus of Healthy Bighead Catfish and Its Efficacy Against Several Fish Pathogens

**DOI:** 10.3390/microorganisms7110549

**Published:** 2019-11-10

**Authors:** Anurak Bunnoy, Uthairat Na-Nakorn, Pattanapon Kayansamruaj, Prapansak Srisapoome

**Affiliations:** 1 Laboratory of Aquatic Animal Health Management, Department of Aquaculture, Faculty of Fisheries, Kasetsart University, 50 Paholayothin Rd, Ladyao, Chatuchak, Bangkok 10900, Thailand; anurak.bunnoy@gmail.com (A.B.); pattanapon.k@ku.ac.th (P.K.); 2 Laboratory of Aquatic Animal Genetics, Department of Aquaculture, Faculty of Fisheries, Kasetsart University, 50 Paholayothin Rd, Ladyao, Chatuchak, Bangkok 10900, Thailand; ffisurn@ku.ac.th

**Keywords:** *Acinetobacter*, *16S rRNA*, gyrB, *rpoB*, genome sequence, ANI, *in silico* DDH, Clarias microcephalus, probiotics, antagonists, Acb complex

## Abstract

The bacterial strain KU011TH was isolated from the skin mucus of healthy bighead catfish. The strain is a Gram-negative coccobacillus that is nonmotile, aerobic, catalase positive, oxidase negative, and nonhemolytic. Sequence analyses of the housekeeping genes *16S rRNA*, *gyrB* and *rpoB* indicate that this strain is a new member of the Acb complex of the genus *Acinetobacter* and is closely related to *Acinetobacter pittii* and *Acinetobacter lactucae*. In addition, the genome relatedness-associated ANIb (<95–96%) and *in silico* DDH (<70%) values clearly supported the new member of the genus *Acinetobacter* and the Acb complex. The genome of the strain KU011TH was approximately 3.79 Mbp in size, comprising 3619 predicted genes, and the DNA G+C content was 38.56 mol%. The major cellular fatty acids were C18:1ω9c, C16:0, C16:1, C20:2, C18:2ω6c and C18:1ω9t. The whole-genome sequences and phenotypic, phylogenetic, and chemotaxonomic data clearly support the classification of the strain KU011TH as a new member in the genus *Acinetobacter* which is closest to *A. pittii*. Additionally, the new bacterial strain exhibited strong activity against a broad range of freshwater fish pathogens in vitro.

## 1. Introduction

The genus *Acinetobacter*, belonging to the family Moraxellaceae, was first established by Brisou & Prevot [1] with the type species *Acinetobacter lwoffii*. This genus currently includes 61 published valid species names, including four pairs of synonyms (https://apps.szu.cz/anemec/Classification.pdf, 26 August 2019). Bacteria of the genus *Acinetobacter* are characteristically Gram-negative, nonmotile, catalase-positive, oxidase-negative, coccobacillus aerobes that survive under a wide range of environmental conditions. Representatives of the genus *Acinetobacter* have DNA G+C levels ranging from 34.9 to 47.0 mol% [2], and C18:1ω9c and C16:0 are the major cellular fatty acids [3]. Despite being described as nonmotile bacteria, *Acinetobacter* spp. possess different forms of motility, such as twitching movement due to the pilus features on their cell membrane [4].

The genus *Acinetobacter* is a large and diverse group of biochemically, physiologically and naturally multitalented bacteria. Seven *Acinetobacter* species with valid published names, namely, *A. beijerinckii*, *A. gyllenbergii*, *A. haemolyticus*, *A. junii*, *A. parvus*, *A. tjernbergiae* and *A. venetianus*, belong to hemolytic clades. These bacteria have hemolytic characteristics and can lyse mammalian red blood cells [5,6,7,8]. Moreover, the *Acinetobacter calcoaceticus*-*Acinetobacter baumannii* (Acb) complex is also an important group of bacteria in the genus *Acinetobacter*; these bacteria were isolated from a wide range of environmental habitats and are symptomatically associated with human health. These bacteria currently include six different valid published species, namely, *A. calcoaceticus, A. baumannii, A. pittii, A. nosocomialis, A. seifertii* and *A. lactucae* (a later heterotypic synonym of *A. dijkshoorniae)* [9,10]. The major characteristics of the Acb complex are genetically and physiologically highly similar to each other and difficult to distinguish at the species level by standard methods [11]. However, these species differ in their epidemiology, antibiotic resistance and pathogenicity [12]. Some members of the Acb complex, especially *A. baumannii,* are typically mentioned among Acb complex members associated with opportunistic pathogens in humans with the emergence of antibiotic resistance [11,13,14]. In addition, *A. pittii, A. nosocomialis, A. seifertii* and *A. lactucae* are ubiquitous and widespread and are occasionally associated with emerging important nosocomial pathogens involved in hospital-acquired infections [9,10,15]. During the last decade, some Acb complex and non-Acb complex members have been reported as septicemia pathogens that cause mass mortality in aquatic animals such as fishes, including mandarin fish (*Siniperca chuatsi*) [13], channel catfish (*Ietalurus punetaus*) [14], hybrid Prussian carp (*Carassais auratus gibebio*) [16], and loach (*Misgurnus anguillicaudatus*) [15].

Although most of the members of the genus *Acinetobacter* have been isolated from human clinical specimens and reported as important opportunistic pathogens, the characterization of new members from nonhuman environments and of their benefits to the environment and animals has never been performed. Recently, many novel species of the genus *Acinetobacter* have been thoroughly characterized after being isolated from natural sources, such as honey bees, horses, cotton, rice, bark from *Populus* x *euramericana* cankers, vegetables, activated sludge, soil, river water and other environmental sources worldwide [10,17,18,19,20,21,22,23,24].

To our knowledge, catfish farming in Thailand has substantially increased in recent decades. The catfish industry not only has annually helped increase the national income of Thailand but also has produced an affordable source of animal proteins for consumers in the country and around the world. The annual production of catfish is the second highest in the Thai fish aquaculture industry, after Nile tilapia (*Oreochromis niloticus*), with approximately 76,000 million tons produced in 2000 and a peak production of 159,314 million tons in 2004. Since 2004, the decline in Thai catfish production has averaged 5.76% annually [25]. The native bighead catfish (*Clarias macrocephalus* Günther, 1864) is an important catfish species in the aquaculture industry in Thailand. Because of its unique meat composition, bighead catfish has been used as a brood stock for producing hybrid catfish (*C. macrocephalus × C. gariepinus*). Unfortunately, this species is slow growing, produces limited numbers of fry, is susceptible to infectious diseases and is sensitive to various environmental conditions [26]. Specifically, this species is prone to outbreaks of diseases caused by pathogenic bacteria, especially *Aeromonas hydrophila* and *Flavobacterium columnare*, which has become a major problem contributing to the declining catfish production in Thailand. Traditionally, chemicals and antibiotics have been widely applied to treat diseases in cultured catfish. However, the adverse effects of these substances have been a cause for concern for many years since excessive usage of antibiotics and chemicals can lead to the generation of drug-resistant bacteria and to food and environmental contamination [27]. Alternatively, beneficial and effective microorganisms known as “probiotics” have been introduced into a number of aquatic animal species in the aquaculture industry to control infectious bacterial diseases and enhance growth and immune responses [28]. However, a promising bacterial species originally cultured from Thai catfish has never been described and assessed for use in the catfish industry.

Here, we describe the characteristics of a new bacterial strain of the genus *Acinetobacter*, strain KU011TH. The bacterial strain KU011TH was isolated from the skin mucus of healthy bighead catfish in catfish farms in Pathum Thani Province, Thailand. The complete taxonomic characterization of the bacterial strain supported the classification of the bacterial strain KU011TH as a new member of the genus *Acinetobacter* and the Acb complex, and the name *Acinetobacter* sp. KU011TH is proposed for this strain with the type strain is KU011TH. Furthermore, the obtained data on this novel bacterial strain are necessary for further study of its function and antagonistic efficacy against various fish pathogens that cause harmful diseases in bighead catfish (*C. macrocephalus*) and other fish species. The wide-ranging inhibitory effects of the novel bacterial strain warrant further research to identify effective strategies for its use in disease prevention. The new preliminary research findings on this novel bacterial strain could be expanded upon for the development of a potential probiotic approach to help sustain the catfish aquaculture industry.

## 2. Materials and Methods

### 2.1. Bacterial Isolation and Cultivation

A colony of the bacterial strain KU011TH was isolated from the skin mucus of bighead catfish. The skin mucus was serially diluted 10^−1^ to 10^−4^ in physiological saline (0.85% NaCl), placed on plate count agar (PCA, containing 0.5% tryptone, 0.3% yeast extract, 1.0% dextrose/glucose and 1.5% agar; HiMedia Laboratories) and cultured at 32 °C for 24 h. After incubation, a distinct circular colony with a cream color, an entire edge and a convex shape was subcultured on standard nutrient broth (NB, containing 0.5% tryptone, 0.3% yeast extract and 1.0% dextrose/glucose) for 18–24 h. Bacterial pellets were separated by centrifugation at 3000× *g* for 5 min, suspended in glycerol (20% *v*/*v*) and then preserved at −80 °C. Microscopic examinations of colony and cell morphology were performed to confirm that no contamination by other groups of bacteria occurred. The strain KU011TH was routinely grown on PCA and in NB medium at pH 7.3 and 32 °C for 18–24 h for all phenotypic tests.

This study was carried out in accordance with the principle of the Basel Declaration and the recommendations of the Guide for the Care and Use of Laboratory Animals of the Ethical Committee of Kasetsart University, Thailand, with the approval number ACKU61-FIS-004, approval date: 9 September 2018.

### 2.2. Bacterial Cell Morphology Analysis

Gram staining of bacterial cells grown on PCA or PCA with 1.5% (*w*/*v*) NaCl was performed using light microscopy (Olympus, MA, USA) under 1000× magnification as described in [29]. Bacterial cell size and morphology were analyzed via scanning electron microscopy (SEM) (SU8020, Hitachi High-Technologies, Tokyo, Japan). Briefly, bacterial colonies were grown on PCA plates at 32 °C for 24 h, and thereafter, a single colony was fixed with SEM fixative overnight. The colony samples were dehydrated through an ascending series of ethanol concentrations and then subjected to supercritical drying in a critical point dryer. The samples were sputter coated with gold and inspected by SEM.

### 2.3. DNA Extraction, PCR Amplification and DNA Sequencing

To identify the strain at the species level, the genomic DNA of the bacterial strain KU011TH was obtained using the QIAamp DNA Mini Kit (QIAamp, CA, USA) following the manufacturer’s instructions. DNA was amplified to obtain almost full-length nucleotide sequences of the *16S rRNA* gene using the bacterial universal primers *27f* (5′-AGAGTTTGATCMTGGCTCAG-3′) and *1492r* (5′-TACGGYTACCTTGTTACGACTT-3′) [9,30]. Additionally, parts of the RNA polymerase beta-subunit (*rpoB*) and DNA gyrase subunit B (*gyrB*) genes were also amplified with the *rpoB-696f* (5′-TAYCGYAAAGAYTTGAAAGAAG-3′), *rpoB*-1598r (5′-CGBGCRTGCATTTGTCRT-3′), *gyrBf* (5′-CAGGAAACAGCTATGACCAYGSNGGNGGN AARTTYRA-3′) and *gyrBr* (5′-TGTAAAACGACGGCCAGTGCNGGRTCYTTYTCYTG RCA-3′) primers, which have been previously described [31,32], to verify the genotypic similarities between the novel species and the other members of the genus. The PCRs were performed using Phusion high-fidelity DNA polymerase with proofreading activity (Thermo Fisher Scientific, Waltham, MA, USA) according to the manufacturer’s instructions. The PCR was initiated with a predenaturation step at 95 °C for 5 min, which was followed by 30 cycles of 95 °C for 30 s, 55 °C for 30 s, and 72 °C for 2 min and then finished with postextension at 72 °C for 10 min. The PCR products were purified and cloned into the CloneJET1.2/blunt vector (Thermo Fisher Scientific, MA, USA), and the constructs were sequenced by the Macrogen sequencing service (Macrogen Inc., Seoul, Korea) using the pJET1.2 forward and reverse sequencing primers.

### 2.4. Phylogenetic Analysis

The sequences of the *16S rRNA*, *gyrB* and *rpoB* genes were BLASTed (https://blast.ncbi.nlm.nih.gov/Blast.cgi) against the latest release of the GenBank database. Similarity calculations for the obtained sequences were performed using MATGAT version 2.0 software [33]. Additionally, the DNA sequences were aligned with those of related *Acinetobacter* species using ClustalW [34]. Phylogenetic trees were constructed using Molecular Evolutionary Genetics Analysis (MEGA) software, version 7.0 (Proprietary freeware, Japan) [35], with neighbor-joining (NJ) [36] algorithms with a bootstrap of 1000 replications.

### 2.5. Genome Sequencing, Assembly and Annotation

The quality of the extracted DNA was determined using QubitTM fluorometric quantitation (Thermo Fisher Scientific, USA). Preparation of the DNA sequencing library was carried out using Nextera XT kits (Illumina, San Diego, CA, USA), and whole-genome sequencing was performed subsequently using paired-end runs on an Illumina HiSeq platform with a 251-bp read length. Library preparation and whole-genome sequencing were performed by a service provider (Novogene, Singapore). Raw data were then trimmed to filter out low-quality (Q score < 30) and contaminating reads prior to de novo assembly using the A5-miseq pipeline, version MCS 2.3 [37]. The quality of the derived assemblies was determined using the QUAST program [38]. Only contigs with lengths >1000 bp were used for further genome annotation using the MicroScope web-based service (http://www.genoscope.cns.fr/agc/microscope) [39].

### 2.6. Genome Sequencing Analysis, ANI, in silico DDH Calculations and Phylogeny

Bacterial genomic relatedness was investigated by different algorithms for genome-to-genome comparison. The average nucleotide identity (ANI) values were calculated based on BLAST (ANIb) using the web service JSpeciesWS online server (http://jspecies.ribohost.com/jspeciesws/) [40]. For *in silico* DNA-DNA hybridization (DDH) analysis, the results were obtained from the genome-to-genome distance calculator (GGDC) web service using formula 2 (identities/HSP length) (http://ggdc.dsmz.de) [41]. The recommended species cut-offs were lower than 95–96% for ANIb and 70.0% for *in silico* DDH values [42,43]. The genome BLAST distance phylogeny (GBDP) of the bacterial strain KU011TH, closely related Acb complex species and non-Acb complex species in the genus *Acinetobacter* was examined using the type strain genome server (TYGS) [44].

### 2.7. DNA G+C Content Analysis

The G+C content of the bacterial strain KU011TH was automatically obtained from genome calculations using the ANI calculator and MicroScope web platform [38,39].

### 2.8. GenBank/EMBL/DDBJ Accession Numbers

The nucleotide sequences of the bacterial strain KU011TH have been deposited at DDBJ/EMBL/GenBank under the accession numbers MG372049 for the *16S rRNA* gene, MG950236 for the *gyrB* gene, and MG950238 for the *rpoB* gene. The annotated whole-genome shotgun project has been deposited at DDBJ/ENA/GenBank under the accession number PSSN00000000. The version described in this paper is version PSSN01000000.1.

### 2.9. Phenotypic and Chemotaxonomic Analyses

Cytochrome c oxidase activity was determined as previously described [45]. Catalase activity was analyzed with 3% hydrogen peroxide solution, and the production of oxygen bubbles indicated a positive result. Hemolytic activity was tested on Muller-Hinton (MH) agar containing sterile 10% (*v*/*v*) whole sheep blood. Cell motility and oxidative and fermentative activities were analyzed using Hugh and Leifson’s agar (oxidative-fermentative (OF) basal medium; Merck, Germany) supplemented with 10% (*w*/*v*) D-glucose. Growth tests at different temperatures (4, 25, 30, 37, 41 and 45 °C), pH values (4.0–10.0 at intervals of 1.0 pH unit) and NaCl concentrations (0–10% *w*/*v*) were performed in 10.0 mL of MH broth (HiMedia Laboratories). The growth of the bacterium was measured by determining the absorbance at 600 nm and using standard plate counting techniques at 24-hr intervals for 7 days. Tests for the utilization of other carbon sources were performed using API 50 CHB/E test kits (bioMérieux, France). API 20NE (bioMérieux, France) and API 20E test kits (bioMérieux, France) were also used to determine the enzyme activities of the strain according to the manufacturer’s instructions. To confirm the API phenotypic results, hydrolysis of starch, casein and urea was investigated with standard microbiological assays by incubating the strain on starch agar (HiMedia Laboratories), skim milk (HiMedia Laboratories) and urea agar base (HiMedia Laboratories) supplemented with sterile 40% urea solution, respectively. Lipase activity was tested on tributyrin agar (HiMedia Laboratories) [46], and a clear zone around a colony was interpreted as a positive result. All phenotypic tests were performed in triplicate.

### 2.10. Cellular Fatty Acid Analysis

Bacterial cells were cultivated on MH agar at 32 °C for 24 h. Cellular fatty acids were obtained by saponification, methylation and extraction according to version 6.2 of the Sherlock Microbial Identification (MIDI) System by following the instructions for the system [47]. The cellular fatty acid profiles were analyzed by separation of fatty acid methyl esters by a gas chromatography-based method. Peaks were automatically integrated and calculated using Sherlock MIDI, version 6.2 (MIDI, Inc., Newark, DE, USA) [47,48].

### 2.11. Identification of Antibiotic Resistance Genes

The output genome assembly was used to search for similar antibiotic resistance genes in the Comprehensive Antibiotic Resistance Database (CARD) (https://card.mcmaster.ca/home) [49] using BLAST+ [50]. The results were reported according to only the best hits of the genes from the database.

### 2.12. Antibiotic Susceptibility Test

An antibacterial susceptibility test of the bacterial strain KU011TH was performed using the disk diffusion method [51,52]. The cultured bacterial suspension of 2.0 × 10^8^ CFU/mL was grown and prepared as described above and then swabbed on MH agar plates. Commercial paper antibiotic disks with fixed concentrations were placed on the inoculated agar surface. Eighteen antibiotics obtained from Thermo Scientific^TM^ were used in this study, namely, ampicillin (AMP10), amoxicillin (AML10), ciprofloxacin (CIP5), chloramphenicol (C30), cephalothin (KF30), doxycycline (DO30), erythromycin (E15), enrofloxacin (FNR5), neomycin (N30), novobiocin (NV5), oxytetracycline (OT30), polymyxin B (PB300), spectinomycin (SH25), sulfamethoxazole (SXT25), sulfamethoxazole (RL25), cefoperazone (SCF105), tetracycline (TE30) and trimethoprim (W5). The plates were incubated for 18–24 h at 37 °C prior to determination of the results. After incubation, the clear zones of growth inhibition around each of the antibiotic disks were measured in millimeters. The diameter of the clear zone is related to the susceptibility of the isolate, which was interpreted using the criteria published by the Clinical and Laboratory Standards Institute (CLSI) [52]. The results were qualitatively classified into three categories of susceptibility: susceptible, intermediate, and resistant. The test was performed in three replications.

### 2.13. Antagonism Against Pathogenic Bacteria

The antagonistic activity of the bacterial strain KU011TH against pathogenic bacteria was evaluated using both agar dot-spot and quantitative real-time PCR (qPCR) assays. Ten strains that are pathogenic to freshwater and marine aquatic animals obtained from the Laboratory of Aquatic Animal Health Management, Department of Aquaculture, Faculty of Fisheries, Kasetsart University, Thailand, were used to evaluate antagonistic activity. Six strains of freshwater fish pathogens, namely, *A. hydrophila, F. columnare, Flectobacillus roseus, Streptococcus agalactiae, Staphylococcus warneri*, and *Edwardsiella tarda*, and four strains of marine fish pathogens, namely, *Vibrio alginolyticus, Vibrio harveyi, Vibrio parahaemolyticus* AHPND and *Vibrio vulnificus*, were tested.

Using methods previously described in [53], an agar dot-spot assay was conducted on PCA and PCA with 1.5% (*w*/*v*) NaCl for freshwater and marine fish pathogens, respectively. The individual strains of isolate KU011TH and pathogenic bacteria were first inoculated on PCA or PCA with 1.5% (*w*/*v*) NaCl plates at 32 °C for 18 h. After incubation, the cells of the pathogens were collected and resuspended in phosphate-buffered saline (PBS, pH 7.4). A concentration of approximately 1.0 × 10^6^ CFU/mL was used for this experiment. The bacterial suspension was immediately swabbed on prepared PCA or PCA with 1.5% NaCl plates. Then, the strain KU011TH on PCA plates was dot-spotted on the plates that had previously been swabbed with the respective pathogens. After 18 h of incubation at 32 °C, the diameters of the clear zones of growth inhibition were measured in millimeters. The test was performed thrice on each plate with three replications.

### 2.14. Quantitative Analysis of Bacterial Coculture Assay Results

Quantification of the activity of the bacterial strain KU011TH against ten pathogenic bacteria was conducted by coculturing the bacteria and using qPCR assays. The alteration of the cell morphology of the cocultured bacteria was visualized by light microscopy (Olympus, MA, USA) under 1000× magnification. A single colony of a freshwater or marine pathogenic bacterium was either inoculated in 30 mL of standard NB (containing 0.5% tryptone, 0.3% yeast extract and 1% dextrose/glucose) or NB medium with 1.5% NaCl at 32 °C for 18 h. The strain KU011TH was also inoculated in NB and NB with 1.5% NaCl medium for coculture with freshwater and marine pathogenic bacteria. After incubation, bacterial inoculum was collected, purified by centrifugation at 2500× *g* for 5 min and resuspended in PBS (pH 7.4) to attain a final concentration of 1.0 × 10^5^ CFU/mL. All bacterial strains were further monitored for growth on both single-culture and cocultured plates at 24 h. One milliliter of each pathogenic bacterial suspension (1.0 × 10^5^ CFU/mL) was cocultured with 1.0 mL of the bacterial strain KU011TH in a total culture volume of 50 mL of NB or NB with 1.5% NaCl medium at 32 °C for 24 h. The ratio of the cocultured bacteria was 1:1. The estimated starting number of each bacterial strain was 2.0 × 10^3^ CFU/mL (total starting concentration of 4.0 × 10^3^ CFU/mL of each coculture). For the control of each bacterial strain, 1.0 mL of each bacterial suspension was grown without strain KU011TH in a total culture volume of 50 mL of NB or NB with 1.5% NaCl medium at 32 °C for 24 h, and the growth was measured as a single culture.

At 24 h of cultivation, 1.0 mL aliquots from all cocultures and single cultures (control) of each test were collected to extract genomic DNA for quantifying DNA copy number and determining the proportion of cultured bacteria using a qPCR assay. Bacterial genomic DNA was obtained using the QIAamp DNA Mini Kit (QIAamp, CA, USA) following the manufacturer’s instructions. The primers for the qPCR assay were designed to measure primer specificity and efficiency for individual strains (Table 1).

A qPCR assay was performed with Brilliant III Ultra-Fast SYBR^®^ Green (Agilent, CA, USA) in Mx3005P QPCR Systems (Agilent, Santa Clara, CA, USA). The qPCRs were optimized in 20 µL reaction volumes containing 10 µL of 2 × SYBR Green QPCR Master Mix with 0.5 mM primer. qPCR cycling conditions included an initial cycle of 95 °C for 5 min; 40 cycles of 95 °C for 30 s, 55 °C for 30 s, and 72 °C for 90 s; followed by a final extension at 72 °C for 10 min. The specificity of the primer set was validated against the eleven reference strains. Using the above optimized real-time PCR amplification conditions, a standard curve for quantification of all bacterial strains was prepared using 10-fold DNA serial dilution from 10^2^ to 10^10^ copy numbers/mL. The threshold cycle (*C^T^*) was measured for each qPCR. The calculated *C^T^* values of the samples were then plotted against the numbers of microorganisms compared to the control (single culture). The resulting values (DNA copy numbers) were calculated from the standard equation for each bacterial strain. All tests and qPCRs were performed in triplicate.

The results for the numbers of bacteria are presented as the mean ± standard deviation. Student’s *t*-test was used to analyze the data. A *p* value less than 0.05 was considered statistically significant.

## 3. Results and Discussion

**Figure 1:**
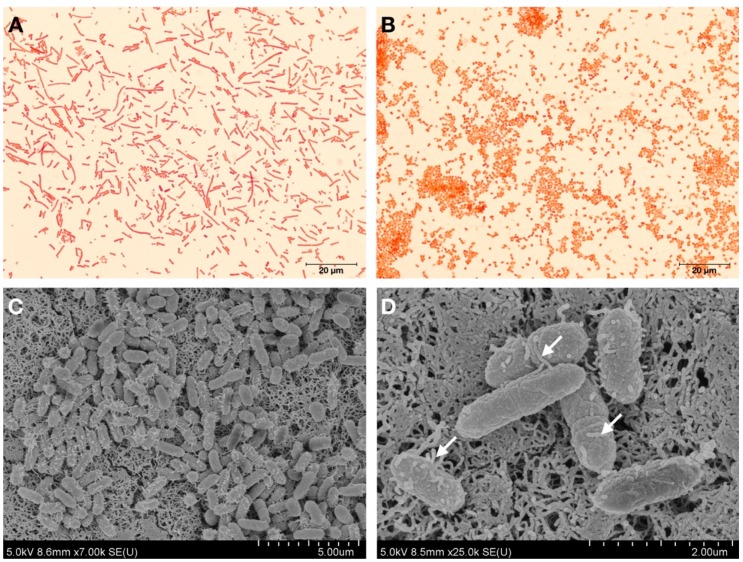
Gram staining and cell morphology observed by light microscopy (100×) of the bacterial strain KU011TH grown on different media: PCA (**A**) and PCA with 1.5% NaCl (**B**). Scanning electron micrographs of the bacterial strain KU011TH (**C**,**D**). Arrows indicate the presence of pili on the bacterial cell wall.

The bacterial strain KU011TH was isolated from the skin mucus of healthy bighead catfish (*C. macrocephalus* Günther, 1864) in Pathum Thani Province, Thailand, on 20 August 2016. Cells from the bacterial strain KU011TH were Gram-negative, non-spore-forming coccobacilli. The cells had various sizes, ranging from 0.8 to 1.5 microns. Pilus structures were found on the cell membranes of the bacterial strain, as shown in Figure 1.

A comparison of the sequences of the housekeeping genes *16S rRNA, gyrB* and *rpoB* showed that the bacterial strain KU011TH clustered with the members of the Acb complex of the genus *Acinetobacter*. The *16S rRNA* sequences of the bacterial strain shared high similarity, ranging from 98.0 to 99.9%, with the sequences from the bacterial strains in the Acb complex. Furthermore, the similarities of the *gyrB* and *rpoB* genes ranged from 87.6 to 97.6% and 87.6 to 98.7%, respectively. Based on sequence analysis of three housekeeping genes, the species most closely related to the bacterial strain KU011TH among the species in the Acb complex were *A. pittii, A. lactucae, A. calcoaceticus, A. nosocomialis, A. seifertii* and *A. baumannii*. Gene sequence similarities between the bacterial strain KU011TH and the most closely related strains in the Acb complex and in the genus *Acinetobacter* are shown in Table 2.

**Figure 2:**
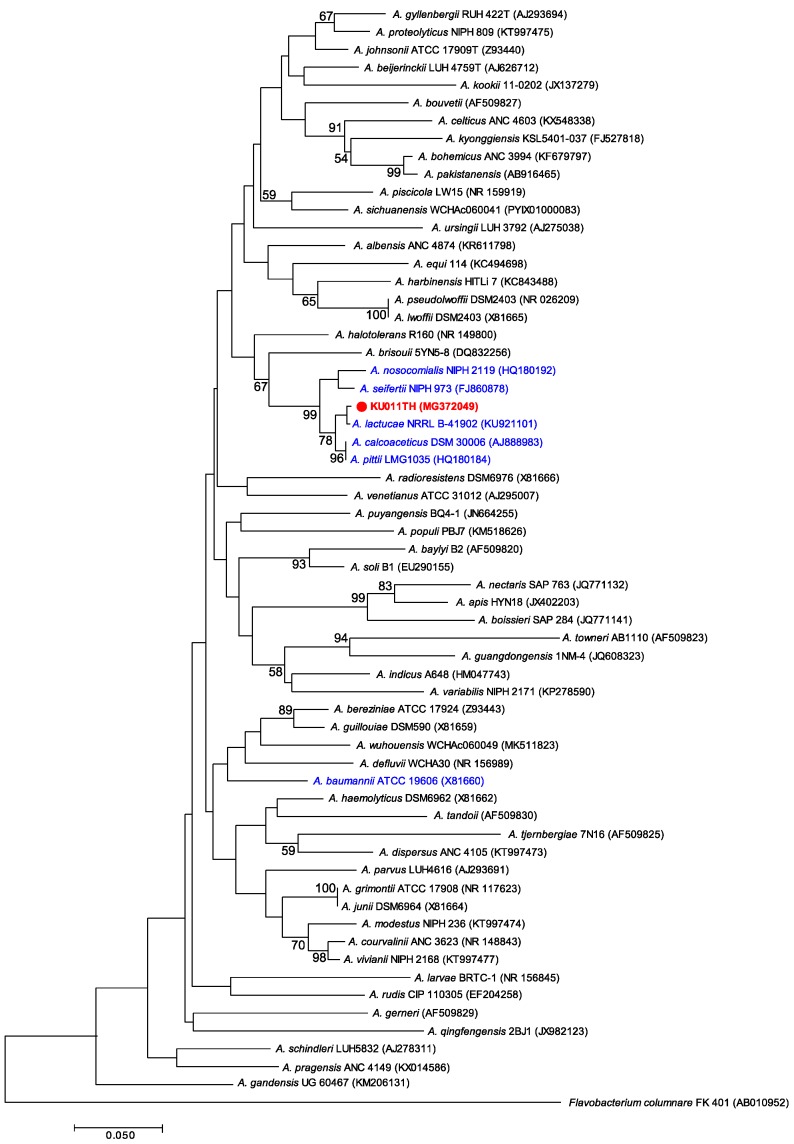
Phylogenetic relationships of the bacterial strain KU011TH (red colored font) and related species in the Acb complex (blue colored font) and other species in the genus *Acinetobacter* based on partial *16S rRNA* gene sequences. Sequence alignments were optimized using ClustalW [34]. Molecular Evolutionary Genetics Analysis (MEGA) software, version 5.0, with the neighbor-joining (NJ) method was used to perform phylogenetic analysis and obtain the phylogenetic tree [36]. Bootstrap values ≥ 50% based on 1000 replications are shown at branch nodes. The *16S rRNA* gene sequence of *F. columnare* FK 401 (AB010952) was used as an outgroup. The GenBank accession number of each strain is given in parentheses.

The phylogenetic tree of the *16S rRNA* gene sequence from the bacterial strain KU011TH and other strains in the Acb complex and non-Acb complex of the genus *Acinetobacter* was constructed and is shown in Figure 2. The *16S rRNA* gene phylogram showed that this strain was a member of the Acb complex and clearly separated from the most closely related bacterial strains in the Acb complex, especially *A. lactucae* (99.9% of *16S rRNA* sequence similarity), *A. pittii* (99.8% of *16S rRNA* sequence similarity) and *A. calcoaceticus* (99.5% of *16S rRNA* sequence similarity). In addition, the phylograms demonstrated that the bacterial strain KU011TH was within the same clade as the Acb complex without *A. baumannii*, as shown in Figure 2. Furthermore, the *16S rRNA* phylogram clearly shows that the bacterial strain KU011TH and several closely related strains are genotypically distinct from the valid published strains in the Acb complex and in the genus *Acinetobacter*.

The species status of the bacterial strain KU011TH in the Acb complex was supported by the genetic relatedness determined by the genome-wide analysis, average nucleotide identity (ANIb) values and *in silico* DDH values. Furthermore, the ANIb and *in silico* DDH values between the bacterial strain KU011TH and Acb complex were 94.0–94.6% and 62.4–63.2%, respectively, for *A. pittii*; 92.2–92.7% and 50.1–51.3%, respectively, for *A. lactucae*; 89.4–90.2% and 38.5–40.8%, respectively, for *A. calcoaceticus*; 87.3–87.6% and 34.1–34.5%, respectively for *A. seifertii*; 87.1–87.3% and 33.7–33.9%, respectively, for *A. baumannii*; and 86.8–87.2% and 33.1–33.4%, respectively, for *A. nosocomialis* (Table 3). Additionally, the calculated *in silico* DDH values were also validated by the differences in percent genomic G+C content between distinct species, which were quite close to zero and not larger than 1.0 [44].

The genetic relatedness values for ANIb and *in silico* DDH proved the species status between the bacterial strain KU011TH and its closest type strain in the Acb complex; the values were considerably lower than the recommended cut-off values for species delineation (95–96% for ANIs and 70% for *in silico* DDH values), confirming our conclusion that the bacterial strain KU011TH represents a novel bacterial species in the Acb complex and in the genus *Acinetobacter*.

**Figure 3:**
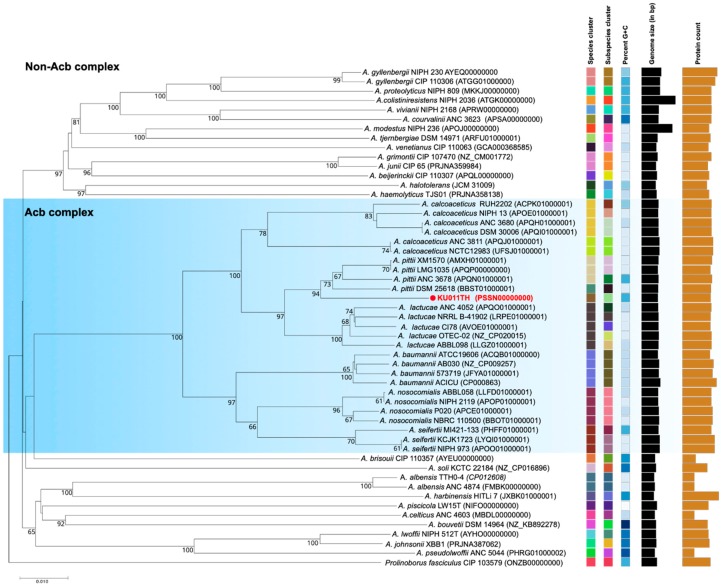
Genome BLAST distance phylogeny (GBDP) showing the position of the bacterial strain KU011TH (red colored font), which is closely related to the Acb complex and non-Acb complex species in the genus *Acinetobacter*. The type (strain) genome server (TYGS) was used to generate GBDP phylograms [44]. Bootstrap values ≥ 50% based on 1000 replications are shown at branch nodes. The genome sequences of *Prolinoborus fasciculus* CIP 103579 (ONZB00000000) were used as an outgroup. The GenBank genome accession number of each strain is given in parentheses.

Phylogenetic analysis based on the core genome of the bacterial strain KU011TH further indicated that the bacterial strain KU011TH belonged to the Acb complex cluster in the same subcluster as *A. pittii*. However, our results indicated that within the Acb complex, the bacterial strain KU011TH is genomically separated from its closely related species (*A. pittii*) as well as from the other non-Acb complex species of the genus *Acinetobacter* with valid published names. The results of the phylogenetic analysis based on the core genome of the Acb complex and non-Acb complex of the genus *Acinetobacter* are shown in Figure 3.

**Figure 4:**
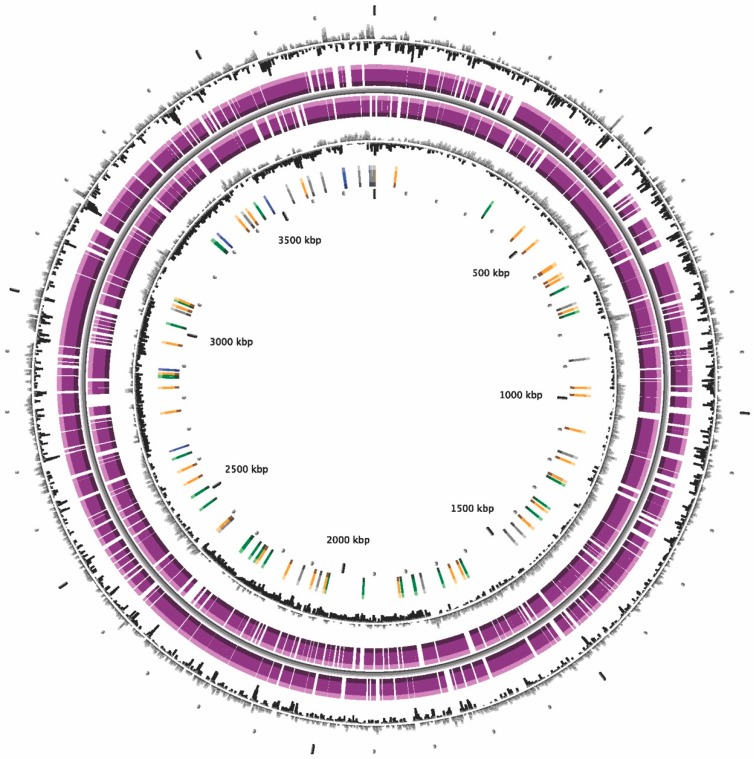
Graphical representation of the circular genome map of the bacterial strain KU011TH. CGView description, from the outside to the center: (1) GC percent deviation (GC window - mean GC) in a 1000-bp window. (2) Predicted CDSs transcribed in the clockwise direction. (3) Predicted CDSs transcribed in the counterclockwise direction. (4) GC skew (G+C/G-C) in a 1000-bp window. (5) rRNA (blue), tRNA (green), misc_RNA (orange), transposable elements (pink) and pseudogenes (gray).

The general genomic characteristics for bacterial strain KU011TH and closely related type strains in the Acb complex are shown in Table 4. In this study, A5-miseq generated 27 contigs from the bacterial strain KU011TH. The genome size of the novel bacterial strain was approximately 3.79 Mbp, and the N50 value was 325. The approximate coverage depth of the assemblies was 172×. According to a MicroScope annotation scheme, the total number of CDSs, rRNAs and tRNAs in strain KU011TH was 3619, 8 and 63, respectively. Additionally, the genomic features and genome distribution pattern of the novel bacterial strain KU011TH are illustrated in Figure 4 and shown in Table 4, respectively. Furthermore, the G+C content of the bacterial strain KU011TH was 38.5 mol%, which is in the range from 34.9 to 47.0 mol% reported for members of the genus *Acinetobacter* and close to the G+C content of species of the Acb complex, including *A. pittii* (38.80 mol%), *A. lactucae* (38.80 mol%), *A. calcoaceticus* (38.70 mol%), *A. nosocomialis* (38.70 mol%), *A. seifertii* (38.60 mol%) and *A. baumannii* (38.90 mol%) (Table 4).

To further evaluate the phenotypic relationship among the species of the Acb complex, metabolic and physiological tests were performed as described [10,54]. The strain was oxidase negative and catalase positive. Motility and hemolytic activity were not observed for the strain. However, the strain was oxidation positive and fermentation negative on OF basal medium. The strain was negative for hydrolysis of starch, casein and urea and positive for lipase activity on tributyrin agar (HiMedia Laboratories). The strain grew at temperatures ranging from 4 to 41 °C, at pH values from 3.0 to 10.0, and in the presence of 0–10% (*w*/*v*) NaCl. Optimal strain growth was observed at 30–32 °C, pH 7.0–8.0 and in the presence of 1.0% (*w*/*v*) NaCl. The differential phenotypic characteristics of the bacterial strain KU011TH and the most closely related strains in the Acb complex of the genus *Acinetobacter* are shown in Table 5.

The predominant fatty acids in the bacterial strain KU011TH were C18:1ω9c (35.3%), C16:0 (31.2%), C16:1 (8.5%), C20:2 (5.2%), C18:2ω6c (3.5%) and C18:1ω9t (3.5%). The different cellular fatty acid profiles of the bacterial strain KU011TH and the most closely related species in the Acb complex and non-Acb complex of the genus *Acinetobacter* are listed in Table 6.

The antibiotic resistance genes in strain KU011TH were analyzed by searching the CARD [49]. The obtained antibiogram of the bacterial strain KU011TH genome exhibited a number of genes that encoded resistance to several antibiotic compounds, including the *acrA, acrB, alaS, ampC, bepE, macB, mfd, nolG, smvA* and *tufB* genes, and is summarized in Table 7.

**Figure 5:**
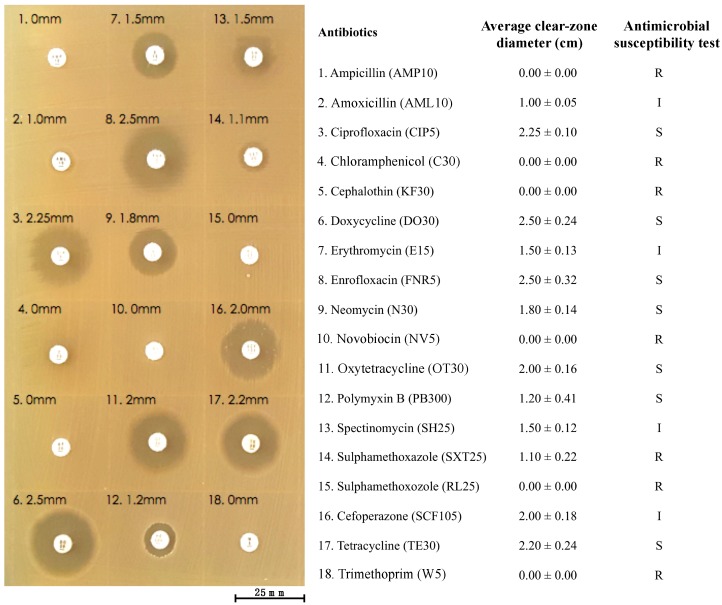
Antibiotic susceptibility of the bacterial strain KU011TH to eighteen commercial antibiotics.

Eighteen commercial antibiotics were used to evaluate the antibiotic susceptibility of the bacterial strain KU011TH. The strain was resistant to seven common antibiotics, namely, ampicillin (AMP10), chloramphenicol (C30), cephalothin (KF30), novobiocin (NV5), sulfamethoxazole (SXT25), sulfamethoxazole (RL25) and trimethoprim (W5). The strain was also completely susceptible to ciprofloxacin (CIP5), doxycycline (DO30), enrofloxacin (FNR5), neomycin (N30), oxytetracycline (OT30), polymyxin B (PB300) and tetracycline (TE30). The strain exhibited intermediate susceptibility to only four antibiotics used in this experiment, namely, amoxicillin (AML10), erythromycin (E15), spectinomycin (SH25) and cefoperazone (SCF105). The levels of resistance or susceptibility to eighteen antibiotics of the strain varied widely (Figure 5). These findings are the first to provide important information regarding the antibiotic resistance levels of the bacterial strain KU011TH. This step is necessary to provide an indication of the potential use of this bacterium in nonclinical or environmental settings.

**Figure 6:**
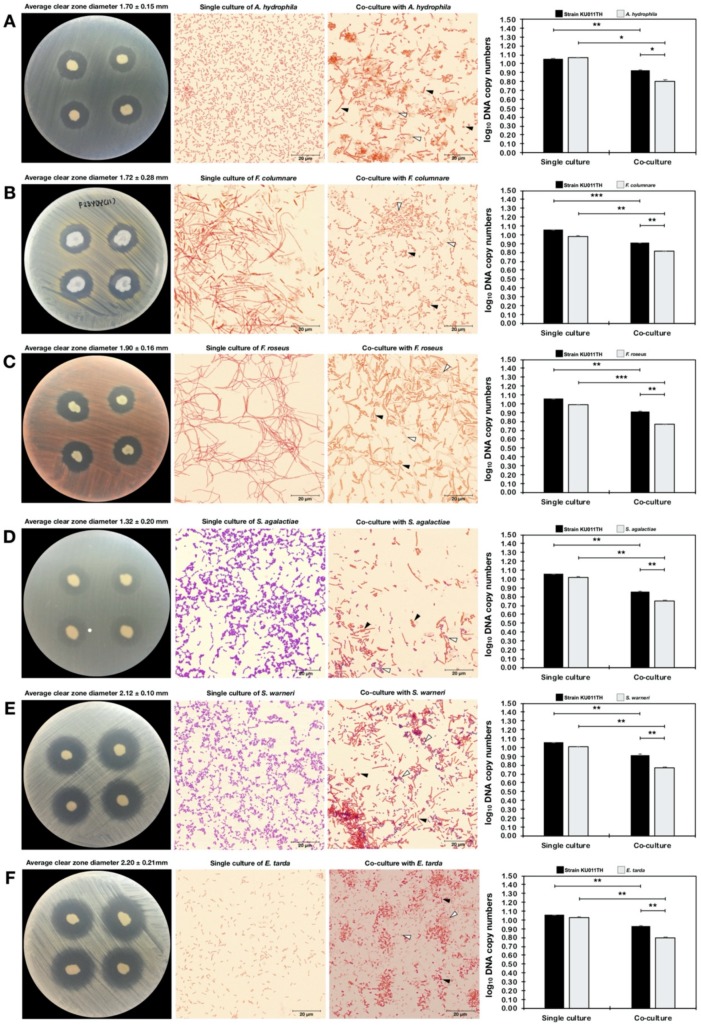

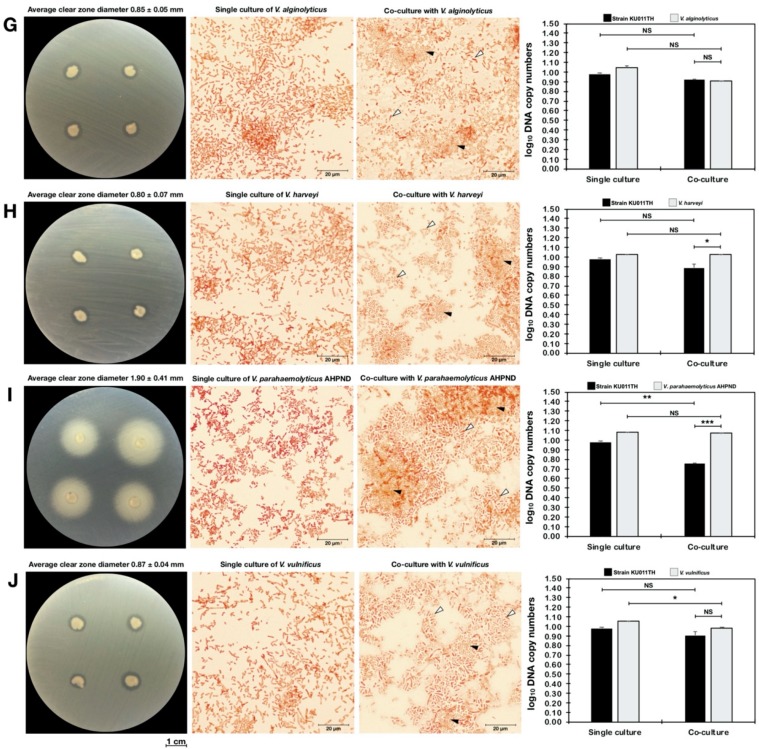
Antagonistic activity of the bacterial strain KU011TH against pathogenic bacteria in freshwater and marine aquatic animals according to agar dot-spot and coculture assays. The results of antagonistic activity are presented as the clear-zone diameters and log_10_ DNA copy numbers of each strain from agar dot-spot and coculture assays, respectively. Interactions between cocultured bacteria were analyzed using Gram staining by light microscopy (100×). The pathogenic bacteria used in the antagonistic activity assay included *A. hydrophila* (**A**), *F. columnare* (**B**), *F. roseus* (**C**), *S. agalactiae* (**D**), *S. warneri* (**E**), *E. tarda* (**F**), *V. alginolyticus* (**G**), *V. harveyi* (**H**), *V. parahaemolyticus* AHPND (**I**) and *V. vulnificus* (**J**). Bacterial DNA copy numbers of single culture and cocultures of each strain were quantified by a qPCR assay using specific primers. Superscripts in the coculture results indicate the levels of significant difference compared to the control (single culture) of each strain using Student’s *t*-test. For strain KU011TH, the single cultures in NB medium and NB medium with 1.5% NaCl were used as the control for cocultures with freshwater or marine fish pathogens, respectively. Black and white arrows indicate the cell morphology of the bacterial strain KU011TH and pathogens during the coculture assay at 24 h, respectively. NS, not significantly different; * = *p*< 0.05, ** = *p*< 0.01 and *** = *p*< 0.001.

Because the bacterial strain KU011TH was originally isolated from the skin mucus of healthy bighead catfish, its antagonistic activity against pathogenic bacteria in freshwater and marine aquatic animals was examined to promote and apply the strain as a probiotic for sustainable aquaculture in the future. The strain exhibited strong antagonistic activities against all freshwater fish pathogens, including A. *hydrophila, F. columnare, F. roseus, S. agalactiae, S. warneri* and *E. tarda*, in agar dot-spot assays. Interestingly, the strain could also grow in saline conditions and exhibited activity against marine fish pathogens, including *V. alginolyticus, V. harveyi, V. parahaemolyticus* AHPND and V. vulnificus, in agar dot-spot assays. The levels of antagonistic activity against all pathogenic bacteria in agar dot-spot assays are shown in Table 8 and Figure 6.

To confirm the predominant antagonistic activities of the novel bacterial strain toward various fish pathogens, growth inhibition through interactions between strain KU011TH and pathogens in coculture conditions was further addressed. The inhibitory effects of the bacterial strain KU011TH on pathogen growth were strongly observed in freshwater fish pathogens. In particular, coculture significantly decreased the growth of *A. hydrophila* (*p* < 0.05), *F. columnare* (*p* < 0.01), *F. roseus* (*p* < 0.01), *S. agalactiae*, *S. warneri* (*p* < 0.01) and *E. tarda* (*p* < 0.01) compared to their growth in single cultures (Table 8). Changes in the cell morphology of cocultured bacteria were observed for *F. columnare*, *A. hydrophila* and *E. tarda,* which exhibited fragile cells and accumulation of inactive cells during coculture compared with their single cultures (Figure 6).

In contrast, growth was not significantly decreased in cocultures of the novel probiotic strain KU011TH and marine fish pathogens, including *V. alginolyticus, V. harveyi, V. parahaemolyticus* AHPND and *V. vulnificus*, compared with the corresponding single cultures (*p* > 0.05) (Table 8). Additionally, significantly decreased growth of the bacterial strain KU011TH in coculture with *Vibrio* spp. compared to the growth in single culture was not observed (*p* > 0.05), whereas significantly decreased growth of the bacterial strain KU011TH in coculture with *V. parahaemolyticus* AHPND was observed (*p* < 0.01). No changes in the cell morphology of the strain in coculture with *Vibrio* spp. were observed. Full-scale interactions indicating the antagonistic activity of the bacterial strain KU011TH against various fish pathogens are shown in Table 8 and Figure 6.

In aquaculture systems worldwide, especially in Thailand, disease outbreaks continue to be a major problem for sustainable development of the aquaculture industry. This issue can negatively impact fish health and economic gain. A number of bacterial species in aquatic environments are capable of causing infectious diseases in various species of freshwater and marine aquatic animals. These pathogens include *A. hydrophila* (aeromonad septicemia), *F. roseus, F. columnare* (columnaris disease), *S. agalactiae* (streptococcosis), *S. warneri* (staphylococcosis), *E. tarda* (edwardsiellosis), *V. alginolyticus* (vibriosis), *V. harveyi* (luminescent vibriosis in shrimps), *V. parahaemolyticus* AHPND (acute hepatopancreatic necrosis disease) and *V. vulnificus* (vibriosis). Traditionally, the use of various chemicals, such as pesticides, disinfectants, and antibiotics, is extensive in the treatment of diseases in cultured fishes. However, the adverse effects of these chemicals have been a cause for concern for many years because excessive usage of antibiotics and chemicals can lead to the generation of drug-resistant pathogens as well as to food and environmental contamination [27,53].

The use of probiotics has increased dramatically, with applications in fields ranging from human health care to the aquaculture industry. Consequently, the development of probiotics as an alternative strategy to prevent fish diseases has received increasing attention in the fish farming industry. Probiotics are defined as “live microorganisms which have a beneficial effect to the host” by increasing growth performance, improving nutrient digestion, enhancing immune responses and enriching the quality of the aquatic environment [60]. Common probiotics used in the aquaculture industry include *Aeromonas, Bacillus, Enterococcus, Enterobacter, Lactobacillus, Lactococcus, Pseudomonas*, and *Vibrio* species [61]. However, microorganisms with probiotic efficiency originating from the genus *Acinetobacter* have not been reported to date.

Although the mechanisms underlying the antagonism of probiotics against aquatic pathogens have not been well documented, several studies revealed that most probiotics can produce several antimicrobial compounds, which play a key role in the antagonistic activities against pathogens. As an antagonist against pathogenic bacteria, the bacterial strain KU011TH produced clear zones free of pathogens, demonstrating that the secretion of antimicrobial compounds from the bacterial strain KU011TH could inhibit the growth of different freshwater fish pathogens in coculture conditions. In similar results, the supernatant from the culture medium of *Bacillus* spp. showed strong inhibitory effects on *S. typhimurium, L. monocytogenes, S. aureus, V. vulnificus, V. harveyi, E. coli* and *S. aureus* [62]. Previous research has also revealed that *Bacillus* species can produce various antimicrobial compounds, such as bacteriocin, surfactins, iturin, fengycin, bacilysin, subtilin, and sublancin, some of which have been widely used to control microorganisms in the food industry [63,64]. Additionally, lipopeptide N3 and amicoumacin A are produced by *B. amyloliquefaciens* and *B. pumilus* and exhibit strong anti-*Vibrio* activities, disrupting cell membranes and causing cell lysis [65]. Moreover, lactic acid bacteria (LAB) species can produce a number of antimicrobial compounds, such as hydrogen peroxide and bacteriocin-like substances, which have strong inhibitory activities against the pathogens *V. metschnikovi, V. harveyi* and *S. aureus*, which infect orange-spotted grouper [66]. Recently, several anti-A. salmonicida compounds isolated from *B. velezensis* V4 belonging to the iturin, macrolactin, and difficidin groups were identified by [67]. These compounds strongly inhibited the growth of *A. salmonicida in vitro*. However, the antimicrobial mechanisms of the bacterial strain KU011TH on freshwater fish pathogens could not be directly explained, suggesting that some antimicrobial peptides produced by the bacterial strain KU011TH are able to either kill pathogens or inhibit their modes of action. Despite the inactivity of the bacterial strain KU011TH against marine fish pathogens in coculture conditions, this strain showed the ability to grow in culture with *Vibrio* spp. under the same conditions, suggesting that the strain can grow under high-saline conditions but cannot produce antimicrobial compounds and/or antimicrobial molecules and is thus ineffective against pathogens under these conditions. Further work on the purification and identification of the antimicrobial molecules from the bacterial strain KU011TH will help elucidate the mechanism underlying its antimicrobial activity against various fish pathogens.

## 4. Conclusions

Data from phenotypic, phylogenetic, chemotaxonomic and whole-genome sequence analyses strongly support the classification of the bacterial strain KU011TH as a new member in the Acb complex and in the genus *Acinetobacter* which is closest to *A. pittii*. Our findings revealed that the new bacterial strain KU011TH has strong antagonistic activity against various fish pathogens and is the first strain suggested as a potential probiotic from the genus *Acinetobacter*. Thus, the results from the present study strongly suggest that the novel bacterial strain is worth evaluating for further study of its function and efficacy to support disease resistance in the host, bighead catfish (*C. macrocephalus*). This novel bacterial strain is the first potential probiotic believed to be safe for host and human health that could further replace the use of antibiotics in catfish farming.

## Figures and Tables

**Table 1:** Specific primers for qPCR-based bacterial quantification in single and coculture assays of the bacterial strain KU011TH and pathogenic bacteria.

Bacterial Strains	Primer Names	Nucleotide Sequence (5′->3′)	Amplicon Size (bp)	Accession Number
*Acinetobacter* sp. KU011TH	KU011TH_*F* KU011TH_*R*	GGCGTGCGTATTGTTTTACGTGAT CAATACCGTTTTCTGTATCTGCGG	154	MG950236
*Aeromonas hydrophila*	*Aeromonas*_F *Aeromonas*_R	CAAGGCTGATATCTCCTATCCCTATG GCCACTCAGGGTCAGGTCAT	66	KU196733
*Flavobacterium columnare*	*Flavobac*_F *Flavobac*_R	CCTGTACCTAATTGGGGAAAAGAGG GCGGTTATGGCCTTGTTTATCATAGA	113	CP018912
*Flectobacillus roseus*	*Flectoba*_F *Flectoba*_R	AGGGTAGCTACCAGGCAACTGG ATCCCGTTCTTGACGCGGAAC	202	MG322214
*Streptococcus agalactiae*	*Strepto*_F *Strepto*_R	GGAAACCTGCCATTTGCGTCT AATCTATTTCTAGATCGTGGAAT	190	CP033822
*Staphylococcus warneri*	*Staphylo*_F *Staphylo*_F	TGTAGCTAACTTAGATAGTGTTCCTTCT CCGCCACCGTTATTTCTT	62	CP033098
*Edwardsiella tarda*	*Edward*_F *Edward* _R	CAGTGATAAAAAGGGGTGGA CTACACAGCAACGACAACG	114	CP023706
*Vibrio alginolyticus* *Vibrio harveyi* *Vibrio parahaemolyticus* *Vibrio parahaemolyticus* AHPND *Vibrio vulnificus*	*Vibrio*_F *Vibrio*_R	GGCGTAAAGCGCATGCAGGT GAAATTCTACCCCCCTCTACAG	120	GQ455007 * GQ455008 * HQ123986 * NR117907 *

* The designed primers were based on the *16S rRNA* genes of various *Vibrio* spp. in the NCBI database under accession numbers GQ455007, GQ455008, HQ123986, and NR117907 to evaluate all *Vibrio* bacterial strains.

**Table 2:** Sequence similarity and origin of the bacterial strain KU011TH and the most closely related type strain in the genus *Acinetobacter*.

Bacterial Strain	Sequence Similarity with Strain KU011TH (%)			Origin of Bacterial Isolate
	*16S rRNA* Gene	*gyrB* Gene	*rpoB* Gene	
Acb complex				
*A. lactucae* NRRL B-41902 ^T^	99.9	91.2	96.7	Iceberg lettuce, water
*A. pittii* LMG1035 ^T^	99.8	97.6	98.7	Human, soil, water
*A. calcoaceticus* DSM 30006 ^T^	99.5	90.4	93.9	Human, soil, water
*A. nosocomialis* NIPH2119	99.2	89.1	93.1	Human
*A. seifertii* NIPH973 ^T^	99.1	NR	87.6	Clinical specimen
*A. baumannii* ATCC 19606^T^	98.0	87.6	93.6	Clinical specimen
**Non-Acb complex**				
*A. dispersus* ANC 4105 ^T^	98.0	78.7	85.1	Clinical specimen
*A. proteolyticus* NIPH 809 ^T^	98.0	76.4	84.6	Clinical specimen
*A. apis* HYN18 ^T^	97.6	NR	78.2	Tract of a honey bee
*A. albensis* ANC 4874 ^T^	97.5	72.1	81.9	Natural soil and water
*A. vivianii* NIPH 2168 ^T^	97.4	76.4	84.6	Clinical specimen
*A. courvalinii* ANC 3623 ^T^	97.1	76.6	83.3	Conjunctiva (agama lizard)
*A. nectaris* SAP 763 ^T^	97.5	72.4	77.6	Flower (floral nectar)
*A. bohemicus* ANC 3994 ^T^	97.5	54.0	80.3	Natural soil and water
*A. boissieri* SAP 284 ^T^	96.7	69.3	82.9	Flower (floral nectar)
*A. guangdongensis* 1NM-4 ^T^	96.6	71.7	78.7	Abandoned lead–zinc ore
*A. modestus* NIPH 236 ^T^	96.8	78.3	84.1	Urine / clinical specimen
*A. hemolyticus* DSM6962 ^T^	96.5	75.2	81.0	Clinical specimen
*A. pragensis* ANC 4149 ^T^	96.5	68.6	84.1	Natural soil and water
*A. puyangensis* BQ4-1 ^T^	96.7	65.2	73.1	Bark of *Populus* × *euramericana* canker

Percent sequence similarity of *16S RNA*, *gyrB* and *rpoB* genes determined using MATGAT version 2.0 open-source freeware (Department of Biology and Molecular Biology, Montclair State University, New Jersey, USA) [33]. NR, not reported.

**Table 3:** Comparison of the average nucleotide identity (ANIb) and *in silico* DNA-DNA hybridization (DDH) value of the bacterial strain KU011TH and its closest species in the Acb complex.

Bacterial Strain of the Acb Complex	Accession Number	ANIb (%)	*In silico* DDH (%)	G+C Difference (%)
***Acinetobacter pittii***	**Range**	**94.0–94.6**	**62.4–63.2**	**0.05–0.28**
*A. pittii* XM1570	AMXH01000001	94.7	63.2	0.26
*A. pittii* ANC 3678	APQN01000001	94.6	62.4	0.28
*A. pittii* DSM 25618	BBST01000001	94.5	62.7	0.13
*A. pittii* NBRC 110509	BBUA01000001	94.5	63.1	0.05
*A. pittii* LMG1035	NC_016603	94.4	63.1	0.27
*A. pittii* CR12-42	JQNT01000001	94.6	63.1	0.19
***Acinetobacter lactucae***	**range**	**92.2–92.7**	**50.1–51.3**	**0.08–0.28**
*A. lactucae* ANC 4052	APQO01000001	92.2	50.7	0.29
*A. lactucae* CI78	AVOE01000001	92.5	50.5	0.16
*A. lactucae* OTEC-02	NZ_CP020015	92.5	50.5	0.28
*A. lactucae* ABBL098	LLGZ01000001	92.4	50.7	0.27
*A. lactucae* TG29425	RFEL01000001	92.7	51.3	0.09
*A. lactucae* TG41018	RFES01000001	92.6	50.1	0.08
***Acinetobacter calcoaceticus***	**range**	**89.4–90.2**	**38.5–40.8**	**0.01–0.19**
*A. calcoaceticus* RUH2202	ACPK01000001	89.4	38.7	0.03
*A. calcoaceticus* NIPH 13	APOE01000001	89.5	38.9	0.11
*A. calcoaceticus* ANC 3680	APQH01000001	89.4	38.9	0.19
*A. calcoaceticus* DSM 30006	APQI01000001	89.4	38.6	0.09
3.5 *A. calcoaceticus* ANC 3811	APQJ01000001	90.2	40.8	0.01
3.6 *A. calcoaceticus* NCTC12983	UFSJ01000001	89.5	38.5	0.16
***Acinetobacter seifertii***	**range**	**87.3–87.6**	**34.1–34.5**	**0.00–0.09**
*A. seifertii* C917	APCT01000001	87.3	34.1	0.09
*A. seifertii* NIPH 973	APOO01000001	87.4	34.2	0.04
*A. seifertii* KCJK1723	LYQI01000001	87.4	34.3	0.04
*A. seifertii* MI421-133	PHFF01000001	87.4	34.5	0.08
*A. seifertii* KCJK7915	QAYP01000001	87.6	34.3	0.01
*A. seifertii* SAb133	SNSA01000001	87.6	34.3	0.00
***Acinetobacter baumannii***	**range**	**87.1–87.3**	**33.7–33.9**	**0.44–0.51**
*A. baumannii* AB030	NZ_CP009257	87.1	33.8	0.48
*A. baumannii* AB030	CP009257	87.1	33.8	0.48
*A. baumannii* ACICU	CP000863	87.3	33.8	0.47
*A. baumannii* D1279779	CP003967	87.2	33.8	0.44
*A. baumannii* ZW85-1	CP006768	87.3	33.9	0.51
*A. baumannii* XH386	CP010779	87.1	33.7	0.51
***Acinetobacter nosocomialis***	**range**	**86.8–87.2**	**33.1–33.4**	**0.06–0.34**
*A. nosocomialis* P020	APCE01000001	86.9	33.4	0.18
*A. nosocomialis* NBRC 110500	BBOT01000001	86.9	33.3	0.12
*A. nosocomialis* LMG 10619	BBSR01000001	87.1	33.1	0.08
*A. nosocomialis* 6411	NZ_CP010368	87.2	33.3	0.18
*A. nosocomialis* ABBL058	LLFD01000001	86.8	33.3	0.06
*A. nosocomialis* AB6	PXNE01000001	87.0	33.2	0.34

The recommended species delimitation value is lower than 95–96% for ANIb and lower than 70.0% for the *in silico* DDH value [42,43]. ANIb, average nucleotide identity (ANI) based on BLAST; *in silico* DDH, *in silico* DNA-DNA hybridization.

**Table 4:** Genomic characteristics of the bacterial strain KU011TH (accession number PSSN00000000) and other species of the Acb complex species.

Bacterial Strain	Genome Characteristics						
	Accession Number	Genome Size (Mb)	G+C Content (mol%)	Contigs	Coding Sequences (CDS)	rRNA Genes	tRNA Genes
Strain KU011TH	PSSN00000000	3.79	38.56	27	3619	8	63
*A. pittii* LMG1035 ^T^	APQP00000000	3.83	38.80	33	3675	19	78
*A. lactucae* NRRL B-41902^T^	LRPE00000000	3.92	38.60	94	3735	4	63
*A. calcoaceticus* DSM 30006 ^T^	APQI00000000	3.92	38.70	12	3808	18	75
*A. seifertii* NIPH973 ^T^	APOO00000000	4.23	38.60	26	4180	13	70
*A. baumannii* ATCC 19606 ^T^	ACQB01000000	3.93	38.90	2	3725	18	71
*A. nosocomialis* NIPH2119 ^T^	APOP01000001	3.91	38.70	19	3730	18	74

**Table 5:** Differential phenotypic characteristics of the bacterial strain KU011TH and the most closely related strain in the Acb complex of the genus *Acinetobacter*.

Phenotypic Characteristics	Bacterial Strain in the Acb Complex						
	1	2	3	4	5	6	7
Temperature range (°C)	4–41	37–44	15–44	25–44	25–41	37–44	25–41
Temperature optimum (°C)	32	30	30	30	30	37	30
pH range/optimum	3–10/7	NR	6–9/7	NR	NR	NR	NR
NaCl concentration range/optimum (% *w*/*v*)	0–10/1	NR	0–5	NR	NR	NR	NR
Acidification of d-glucose	−	+	−	+	+	+	+
Hemolysis of sheep blood	−	−	NR	−	−	−	−
Adipate/adipic acid	−	+	NR	+	−	+	+
Citrate (Simmons)/trisodium citrate	+	+	NR	+	+	+	+
d-glucose	+	−	NR	−	−	−	−
d-malate/malic acid	−	+	−	−	+	+	+
d-ribose	+	−	NR	−	−	+	+
dl-lactate/d-lactose	−	+	NR	+	+	+	+
Gelatin/gelatinase	−	NR	NR	−	−	NR	NR
l-arabinose	+	+	NR	−	−	+	+
l-arginine	+	+	NR	+	+	+	+
l-sorbose	−	NR	−	NR	NR	NR	NR

The results were either obtained in this study or have been presented previously [10,21]. All data were obtained from the current study using API 50CH, API 20NE, and API 20E. Bacteria were cultured at 30 °C except in the temperature growth tests. The strains were oxidase negative and catalase positive. Motility and hemolytic activity were not observed in the strains. All strains were negative for the hydrolysis of starch, casein, urea and gelatin. Strains: 1, strain KU011TH; 2, *A. pittii* [55], 3, *A. lactucae* [24]; 4, *A. calcoaceticus* [9]; 5, *A. seifertii* [10]; 6, *A. baumannii* [6]; 7, *A. nosocomialis* [55]. +, positive; −, negative; NR, not reported.

**Table 6:** Cellular fatty acid composition (%) of the bacterial strain KU011TH and the reference type strain in the Acb complex and non-Acb complex of the genus *Acinetobacter*. The results were either obtained in this study or have been presented previously [10,19,54]. Strains: 1, strain KU011TH; 2, *A. lactucae* NRRL B-41902 ^T^ [24]; 3, *A. baumannii* LMG 1041 ^T^ [9]; 4, *A. apis* HYN18 ^T^ [56]; 5, *A. indicus* A648 ^T^ [57]; 6, *A. radioresistens* DSM 6976 ^T^ [58]; 7, *A. venetianus* ATCC 31012 ^T^ [8]; 8, *A. parvus* LMG 21765 ^T^ [59]; and 9, *A. junii* LMG 998 ^T^ [6].

Fatty Acid	Bacterial Strain								
	Acb Complex *			Non-Acb Complex					
	1	2	3	4	5	6	7	8	9
C_12:0_	1.8	5.4	9.7	8.3	6.4	11.2	5.1	5.9	3.8
C_14:0_	0.7	-	0.7	3.3	1.7	1.3	0.7	1.7	1.3
C_16:0_	31.2	25.2	17.6	18.0	10.6	15.6	18.2	16.8	16.3
C_17:0_	1.3	1.3	2.4	L	1.5	1.2	2.4	0.7	1.7
C_18:0_	1.8	1.5	0.9	0.5	4.6	1.5	0.7	1.0	2.3
C_16:1_	8.5	-	6.6	-	4.4	2.4	0.4	-	0.4
C_17:1_ω10	0.7	2.0	-	-	-	-	-	-	-
C_18:1_ω9t	3.5	-	-	-	-	-	-	-	-
C_18:1_ω9c	35.4	36.9	34.9	6.1	19.6	25.8	25.2	38.2	28.1
C_18:2_ω6c	3.6	-	-	-	-	15.8	-	-	-
C_20:2_	6.0	-	-	-	-	-	-	-	-

* The cellular fatty acid compositions of some valid published species of the Acb complex, including *A. pittii*, *A. calcoaceticus, A. nosocomialis* and *A. seifertii*, were not reported in the original publications. L, lower than 0.5%; -, not detected.

**Table 7:** Antibiotic resistance genes identified following comparison of the genome sequence of the bacterial strain KU011TH with that of the reference strain in the database.

Gene	Identity (%)	Resistant to
*acrA*	43.10	aminoglycosides, beta-lactams and fluoroquinolones
*acrB*	54.08	panipenem, penems, aztreonam, sulfonamides, azithromycin, novobiocin, meropenem, colistin, ciprofloxacin erythromycin, tetracycline, polymyxin, trimethoprim, aminocoumarin antibiotics, beta-lactams, fluoroquinolones, chloramphenicol, macrolides and tetracycline derivatives
*alaS*	58.72	novobiocin and aminocoumarin antibiotics
*ampC*	93.99	cephalosporins and beta-lactams
*bepE*	97.92	tetracycline, fluoroquinolones and tetracycline derivatives
*macB*	52.72	erythromycin and macrolides
*mfd*	50.61	sparfloxacin, norfloxacin, nalidixic acid, gatifloxacin, moxifloxacin, levofloxacin, ciprofloxacin and fluoroquinolones
*nolG*	30.86	thiamphenicol and chloramphenicol
*smvA*	35.61	fluoroquinolones
*tufB*	68.22	kirromycin and elfamycin

**Table 8:** Growth and antagonistic activity against various fish pathogens of the bacterial strain KU011TH.

Bacterial Strain	Single Culture		Antagonistic Activity of the Bacterial Strain KU011TH Against Pathogens		
			Coculture Assay		Dot-Spot Assay
	DNA Copy Number of Bacterial Cells (copies/mL)		DNA Copy Number of Bacterial Cells (copies/mL)		Clear-Zone Diameter (cm)
	NB Medium	NB Medium with 1.5% NaCl	Strain KU011TH	Pathogen	
Strain KU011TH	2.37 ± 8.81 × 10^11^	3.51 ± 2.14 × 10^9^	-	-	-
*Aeromonas hydrophila*	5.47 ± 3.39 × 10^11^	-	2.85 ± 4.23 × 10^8 **^	2.47 ± 1.54 × 10^6 *^	1.70 ± 0.15
*Flavobacterium columnare*	4.62 ± 6.84 × 10^9^	-	1.30 ± 2.93 × 10^8 ***^	2.97 ± 5.01 × 10^6 **^	1.72 ± 0.28
*Flectobacillus roseus*	6.80 ± 3.23 × 10^9^	-	1.72 ± 1.18 × 10^8 **^	7.00 ± 9.98 × 10^5 ***^	1.90 ± 0.16
*Streptococcus agalactiae*	3.39 ± 5.37 × 10^10^	-	1.45 ± 2.90 × 10^8 **^	4.00 ± 1.39 × 10^5 **^	1.32 ± 0.20
*Staphylococcus warneri*	1.87 ± 1.51 × 10^9^	-	1.59 ± 6.98 × 10^8 **^	8.03 ± 8.64 × 10^5 **^	2.12 ± 0.10
*Edwardsiella tarda*	5.59 ± 3.20 × 10^10^	-	2.80 ± 6.22 × 10^8 **^	1.91 ± 5.38 × 10^6 **^	2.20 ± 0.21
*Vibrio alginolyticus*	-	1.68 ± 3.72 × 10^11^	2.24 ± 3.81 × 10^8 NS^	4.78 ± 6.52 × 10^8 NS^	0.85 ± 0.05
*Vibrio harveyi*	-	3.56 ± 1.23 × 10^10^	8.46 ± 9.78 × 10^7 NS^	4.74 ± 7.58 × 10^10 NS^	0.80 ± 0.07
*Vibrio parahaemolyticus* AHPND	-	1.25 ± 9.69 × 10^12^	5.51 ± 5.13 × 10^5 **^	6.44 ± 1.33 × 10^11 NS^	1.90 ± 0.41
*Vibrio vulnificus*	-	1.72 ± 8.93 × 10^11^	2.10 ± 2.47 × 10^8 NS^	4.23 ± 2.31 × 10^9 *^	0.87 ± 0.04

Bacterial DNA copy numbers of single cultures and cocultures for each strain were quantified by a qPCR assay using specific primers. Superscripts in the coculture results indicate the levels of significant differences compared to the control (single culture) for each strain using Student’s *t*-test. For strain KU011TH, single cultures in NB medium and NB medium with 1.5% (*w*/*v*) NaCl were used as controls for cocultures with freshwater or marine fish pathogens, respectively. NB medium, standard nutrient broth; NS, not significantly different; * = *p* < 0.05, ** = *p* < 0.01 and *** = *p* < 0.001.
